# The bacterial interlocked process ONtology (BiPON): a systemic multi-scale unified representation of biological processes in prokaryotes

**DOI:** 10.1186/s13326-017-0165-6

**Published:** 2017-11-23

**Authors:** Vincent J. Henry, Anne Goelzer, Arnaud Ferré, Stephan Fischer, Marc Dinh, Valentin Loux, Christine Froidevaux, Vincent Fromion

**Affiliations:** 1 Laboratoire de Recherche en Informatique (LRI), UMR 8623, CNRS, Université Paris-Sud/Université Paris-Saclay, Orsay, France; 2 INRA, UR1404, MaIAGE, Université Paris-Saclay, Jouy-en-Josas, France

**Keywords:** Systems biology, Multi-scale systemic description, Prokaryotic biological processes, Mathematical models, Biological ontology

## Abstract

**Background:**

High-throughput technologies produce huge amounts of heterogeneous biological data at all cellular levels. Structuring these data together with biological knowledge is a critical issue in biology and requires integrative tools and methods such as bio-ontologies to extract and share valuable information. In parallel, the development of recent whole-cell models using a systemic cell description opened alternatives for data integration. Integrating a systemic cell description within a bio-ontology would help to progress in whole-cell data integration and modeling synergistically.

**Results:**

We present BiPON, an ontology integrating a multi-scale systemic representation of bacterial cellular processes. BiPON consists in of two sub-ontologies, bioBiPON and modelBiPON. bioBiPON organizes the systemic description of biological information while modelBiPON describes the mathematical models (including parameters) associated with biological processes. bioBiPON and modelBiPON are related using bridge rules on classes during automatic reasoning. Biological processes are thus automatically related to mathematical models. 37% of BiPON classes stem from different well-established bio-ontologies, while the others have been manually defined and curated. Currently, BiPON integrates the main processes involved in bacterial gene expression processes.

**Conclusions:**

BiPON is a proof of concept of the way to combine formally systems biology and bio-ontology. The knowledge formalization is highly flexible and generic. Most of the known cellular processes, new participants or new mathematical models could be inserted in BiPON. Altogether, BiPON opens up promising perspectives for knowledge integration and sharing and can be used by biologists, systems and computational biologists, and the emerging community of whole-cell modeling.

**Electronic supplementary material:**

The online version of this article (10.1186/s13326-017-0165-6) contains supplementary material, which is available to authorized users.

## Background

Systems biology emerged as a promising framework to integrate the whole-cell for different model-organisms [1–3]. However, current cell representations usually refer to specific model organisms, which limits in practice the transfer of whole-cell models to non-model organisms. In contrast, bio-ontologies are a suitable framework for systematically describing biological objects and thus facilitating knowledge transfer among organisms [4, 5]. In this paper, we address the following question: how to combine systems biology and bio-ontology?

Systems biology has its roots in engineering science and conceptualizes the cell as a system composed of interacting sub-systems [1, 6–11]. In this context, cellular processes are typically described as biological subsystems whose inputs (e.g. metabolites, proteins, or sequences, etc.) are converted into outputs by dedicated molecular machines. The molecular machines are usually composed of proteins, consume energy and chemical building blocks, and display a characteristic of operation. This operation can be static or dynamic, deterministic or/and stochastic and is generally described by a formal mathematical model having inputs, outputs and model parameters. For example, a mathematical model can be a nonlinear function or a set of ordinary differential equations. The systemic representation of cells is an efficient framework to interrelate all cellular entities (metabolites, proteins, cellular processes, sequences, etc.), together with their physical or biochemical properties (e.g. kinetic parameters, etc.) [1, 2]. System biologists thus need now an adequate format of systemic description of the whole cell to transfer and share their models. Existing standardized formats for file exchange are adequate to exchange mathematical models for specific cell processes [12, 13], but remain limited to describe a whole-cell model, i.e. a systemic multi-scale representation of interacting complex subsystems.

Bio-ontologies have been developed to formalize and integrate different pieces of biological knowledge [4]. The well-established Gene Ontology (GO) integrates the molecular functions of gene products (GO-MF) with cellular components (GO-CC) and biological processes (GO-BP) [14]. The combined sub-ontologies are commonly used to annotate and characterize gene products [5, 15], but there are also other useful bio-ontologies. The Ontology of Microbial Phenotypes links the phenotypes of bacteria to cellular processes [16]. The Ontology of Genes and Genomes provides a list of genes from different organisms including prokaryotes [17], while the Sequence Ontology (SO) provides a detailed description of polymers and polymer sequence patterns [18]. At another level, the Pathway Ontology (PW) provides a classification of metabolic, signaling and altered eukaryotic pathways [19]. Independently, ChEBI (Chemical Entities of Biological Interest) acts as a reference for the classification of general chemicals according to their chemical structures and modifications [20]. The Systems Biology Ontology (SBO) provides a controlled vocabulary for kinetic parameters and mathematical models of biological processes [21]. Taken together, the existing bio-ontologies cover the concepts necessary to the systemic representation of cells, i.e., biological processes, molecules and mathematical models of biological processes. However, the systemic representation of the whole cell cannot be handled without the addition of further logical relations between existing ontologies.

In this paper, we demonstrates that a systemic multi-scale representation of biological processes, the typical perspective of systems biology, can be formally described as an ontology, and how this ontology can be built based on existing sparse bio-ontologies. As a proof of concept, we developed the Bacterial interlocked Process ONtology (BiPON) and showed that a) heterogeneous biological processes can be described with the systemic representation and b) be linked automatically to mathematical models, and that c) information about these processes can be enriched by automatic reasoning. As a use case, we focus on bacterial gene expression processes, which are well established and representative of known biological processes. They cover, among many other things, combination of polymers, sequence patterns, single molecules or complexes within biological processes, as well as cyclic or branched-point processes. We demonstrated on the use case how a systemic representation of living cells can be formally described and integrated into an ontological model, and what benefits ensue from automatic reasoning on this ontology.

## Methods

### Description of biological processes, corpus building and entity tagging

In the absence of an exhaustive controlled vocabulary in systems biology, we use hereafter the notion of a “biological process”, which comprises the notions of (a) “biological reaction” and “biochemical reaction” as in KEGG (Kyoto Encyclopedia of Genes and Genomes [22]) Reactions database, (b) “biological phenomenon”, “biological pathway” and “biochemical pathway” as in PW or KEGG Pathway database, and finally (c) “biological process” as in GO-BP. Moreover, we use the notion of a “chemical entity” to denote any type of biological compound, including metabolites, proteins, protein complexes, polymers, to cite a few.

**Fig. 1:**
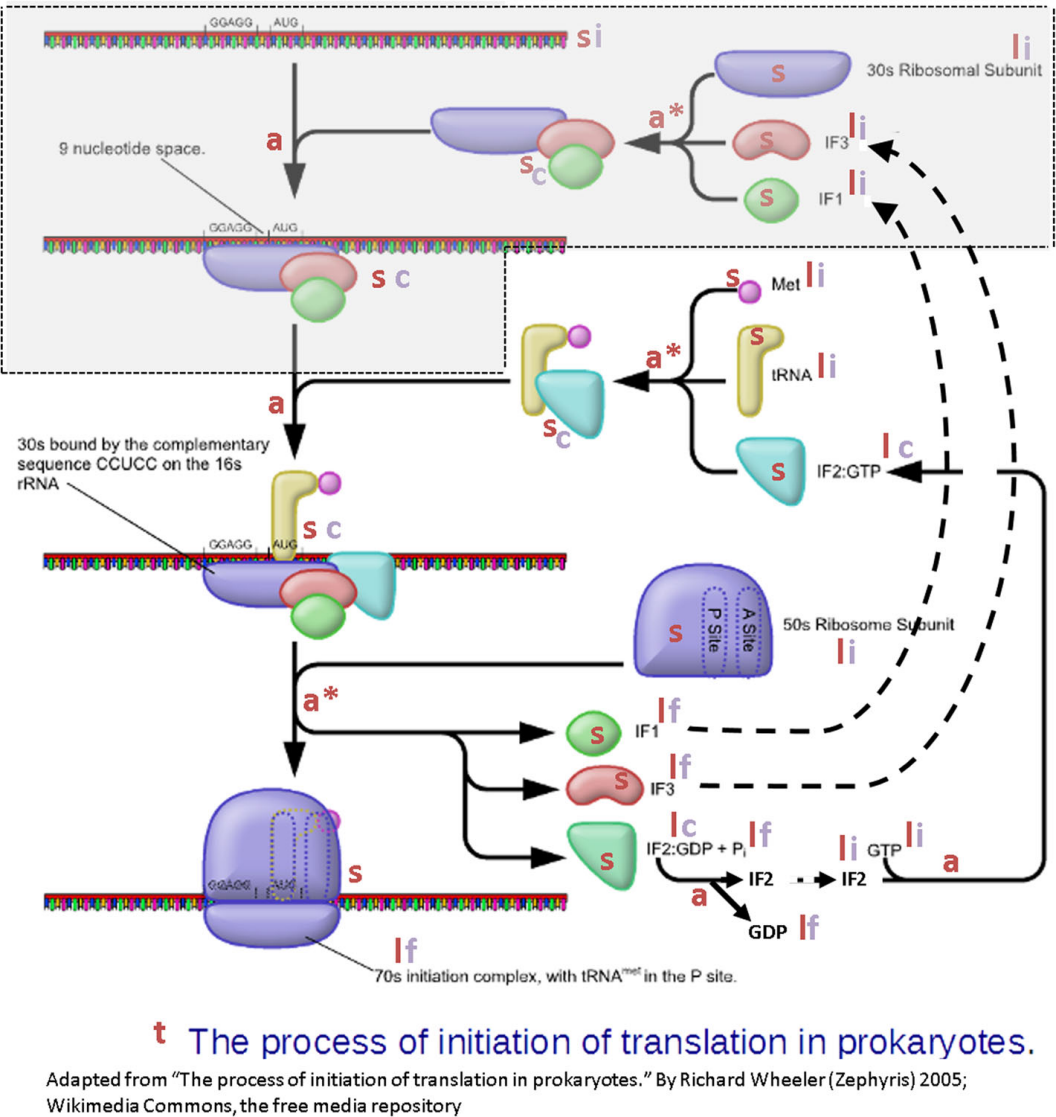
From biological knowledge to systemic description: Identification of the entities of interest for the process of initiation of translation in prokaryotes on a representative figure extracted from Wikimedia. Entities for systemic model design are marked by the following symbols: title (**t**); arrows divided in bifid at origin or head (**a**) or divided in more than two parts (a*) or closing cycle (dotted); shapes (**s**) with legend or label (**l**). Depending on their relative position regarding arrows (origins or heads), three types of biological entities (BioE) are identified (**i**, **f**, **c**): an unframed BioE at arrow-origins (**i**) represents an initial reactant of a process (input); an unframed BioE at arrow-heads (**f**) represents a final product of a process (output); a framed BioE (**c**) represents a product (output) of a process that is the reactant (input) of the next process, and thus that consumed BioE

To develop a dedicated systemic representation for each biological process involved in the bacterial gene expression, we applied the standard state-of-art approach of system engineering. The approach involves two main tasks. (A) We first gathered up-to-date available biological information about the biological process. (B) We then converted the biological information into a systemic representation using boxes, arrows, inputs and outputs, and a mathematical model. We describe and apply below the approach (A) and (B) on a specific example (the formation of the 30S initiation complex) for illustrative purposes. Note that the approach is generic and can be applied on any biological process.

(A) We collected up-to-date knowledge about the biological processes from scientific literature (books, peer-reviewed original articles, and reviews; see Additional file 1 for a list of references). We primarily focused on figures since they facilitate the conversion from biological knowledge to the systemic representation in the task (B) (as illustrated in Fig. 1). Elementary steps composing a biological process are usually found in research articles while books or review articles provide global descriptions of processes. In a few cases, we used figures from didactic web sites and we checked the biological information using original research papers systematically.
(B) We then converted the selected figure into a systemic representation. Despite the heterogeneity of sources, several common features were identified from these schemas (as illustrated Fig. 1): title (**t**), arrows (**a**) and shapes (**s**) with legend or label (**l**).

#### Tagging entities of interest

Given a graphical representation of any biological process with sub-processes (Fig. 1):

- The title (**t**) defines the name of the main biological process that embeds the succession of all identified individual processes.
- The arrows are identified as sub-reactions that correspond to the individual processes. Three types of arrow are distinguished on Fig. 1: linear (**dotted**), bifid at origin or head (**a**) and divided in more than two parts (a*).
- The shapes (**s**) are identified as the chemical entities (BioE) that participate in a biological process and are related to legends or labels (**l**). Depending on their relative position regarding arrows (origins or heads), three types of BioE are identified (**i**, **f**, **c**): an unframed BioE at arrow-origins (**i**) represents an initial reactant of a process (input), called iBioE; an unframed BioE at arrow-heads (**f**) represents a final product of a process (output), called fBioE; a framed BioE (**c**) represents a product of a process (output) which is the reactant of the next process (input), called cBioE (for consumed BioE).

Note that

- Arrows that correspond to BioE recycling within a process are not considered (as illustrated by the dotted arrow in Fig. 1).
- Any BioE may be an initial reactant and/or a product of several distinct processes.

### Biological processes as interlocked systems

After identifying the entities necessary in the biological process, we organized them as a main system composed of different interlocked sub-systems of lower granularity, as follows.

**Fig. 2:**
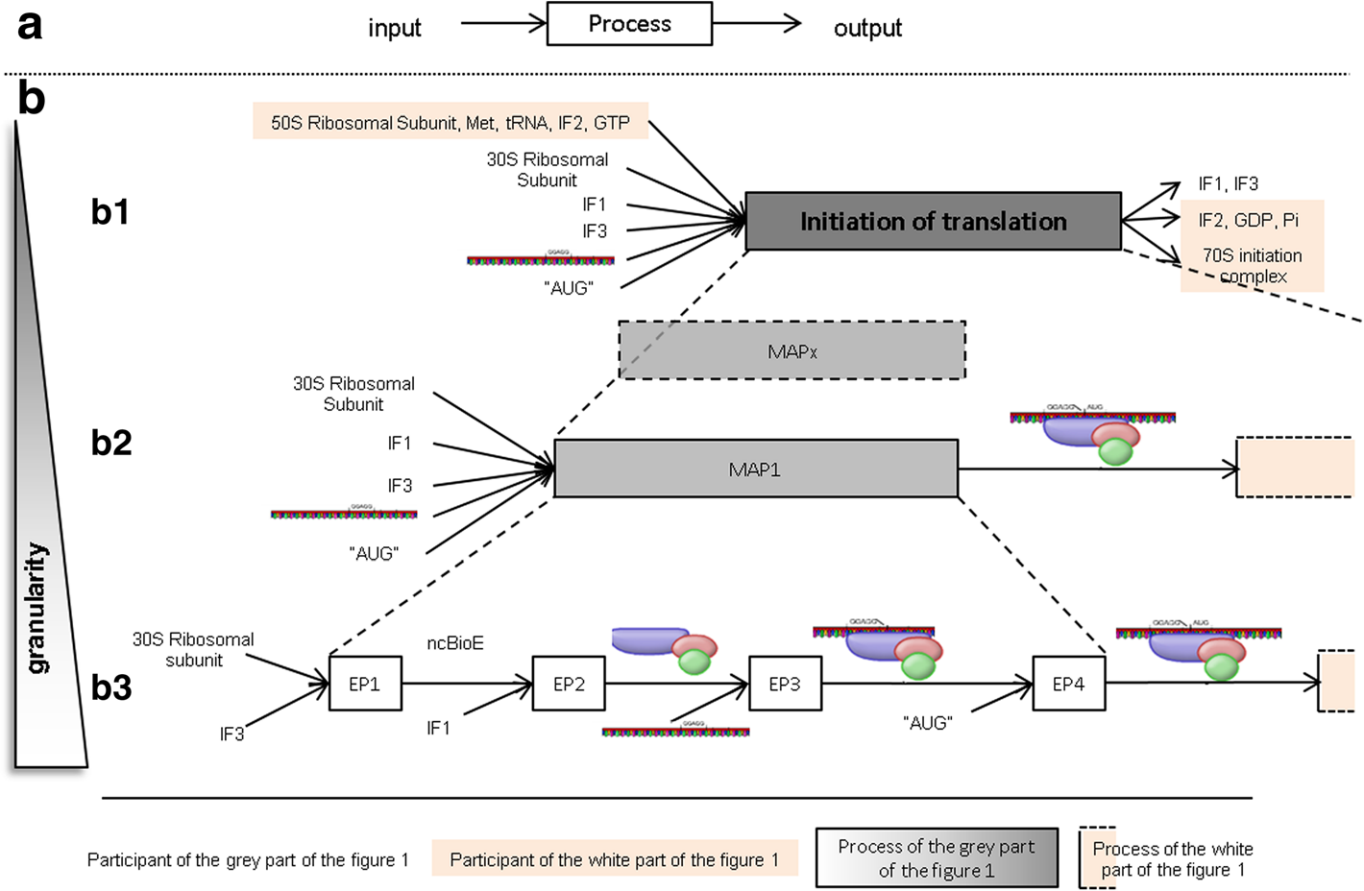
Systemic decomposition of biological processes. **a** Input/output representation of a generic process. **b** Decomposition of a biological process (e.g. initiation of translation) (level B1) into sub-processes of higher granularity, down to the decomposition into elementary processes (level B3). Abbreviations are EP for Elementary Process, MAP for Middle Aggregated Process, ncBioE for next consumed biological entity

An elementary process is formally defined by its participants, i.e. the input(s) and output(s). The standard systemic representation of an elementary process corresponds to a box framed by its input(s)/output(s) (see Fig. 2a). In this graphical representation, inputs are placed on the left of the box at the tail of the incoming arrows, while outputs are placed on the right of the box at the head of the outgoing arrows (Fig. 2a). In our biological context, an elementary process corresponds to a biological reaction and the inputs are the BioEs required for the production of the BioEs that served as outputs.

#### Multi-scale representation of processes

In a multi-scale representation, the same process is represented at different levels of granularity (Fig. 2b). On the top level of granularity, there is a unique aggregated process that leads to output(s) (**B1** in a dark gray box). An aggregated process can be formally defined either by its input(s)/output(s), like an elementary process, or by the composition of successive sub-processes. On the bottom level of granularity, there is a succession of elementary processes that lead to the same output(s) as those produced by other levels (**B3** in white boxes). Via decomposition and aggregation of processes, we can navigate between the different levels of granularity (represented by a gray scale on Fig. 2b).

#### Systemic model of the main process (level B1 on fig. 2b)

The fully aggregated process (at the lowest granularity level) is the main process having iBioEs as inputs and fBioEs as outputs. In the graphics, it is represented by a box and labeled according to the name of the global reaction. The box is framed by BioEs, one iBioE per input of the main process on the left, and one fBioE per output of the main process, on the right.

#### Systemic model of elementary processes (level B3 on fig. 2b)

An elementary process is a sub-reaction of an aggregated process (arrows in Fig. 1), having typically one or two inputs and one or two outputs. In Fig. 1, such a reaction usually concerns bifid arrows (case **a**). In the case of arrows divided into more than two parts (case a* on Fig. 1), and thus implicating at least three inputs or outputs, the process is further split into a sequence of elementary processes through the addition of new consumed BioEs (ncBioE), using additional literature information when available. Two successive elementary processes which share a common participant, i.e. an output of the first elementary process is an input of the second one (cBioE). Elementary processes follow each other until all outputs of the main process are produced (B1 level). Note that cBioEs and ncBioEs never appear as participants in the main process (the fully aggregated one).

#### Systemic model of intermediate processes (level B2 on fig. 2b)

Intermediate processes provide intermediary levels of granularity between the main process and the elementary processes. In the graphical representation, an intermediate process consists of a box that merge boxes of elementary processes. Intermediate processes define sub-processes of specific biological interest. They are built by aggregation of successive elementary processes, following biological considerations, e.g. about the presence of irreversible reactions, the relevance of an intermediate process and of the special nature of a BioE, or the capability to experimentally detect or quantify a specific BioE.

### Mathematical models of biological systems

In systems biology, the community has investigated and developed numerous mathematical models [23, 24] enabling the description, analysis, and simulation of biological processes. Mathematical models can be very different in nature (static, dynamical, stochastic, etc.) and depend on various parameters and variables. One biological process can be described with several mathematical models. For instance, protein translation can be modeled by deterministic [25] or by stochastic models [26]. Conversely, several biological processes can have the same type of mathematical model, such as the Michaelis-Menten equation for the kinetics of different enzymes. In the bio-ontology BiPON, we formalize the relation between biological processes and their mathematical description(s).

### BiPON design

BiPON is a bio-ontology that is composed of two sub-ontologies: bioBiPON and modelBiPON. bioBiPON organizes the systemic description of biological information, while modelBiPON describes the mathematical models associated with biological processes. In the following, a class that has no sub-class for the property *is_a* is called a leaf-class. BiPON has been designed using the software editor Protégé 5 and the Description Logic Manchester syntax [27].

### bioBiPON ontological model

#### Main classes

BioBiPON contains four main classes, which corresponds to the main structure of major bio-ontologies: *Biological process* (GO:0008150), *Chemical entity* (CHEBI:24,431), *Sequence feature* (SO:0000110) and *Cellular component* (GO:0005575).

The classes *Biological process* and C*ellular component* include a selection of GO classes, while the *Chemical entity* class includes a selection of ChEBI classes for small molecules, of SO classes for gene products (e.g. primary transcript), and terms of the KEGG database orthology (KO) for proteins [22]. The *Sequence feature* class includes a selection of SO classes for sequence patterns. Finally, classes which were not present in existing bio-ontologies were created manually.

The *Biological process* class contains as subclasses the biological processes and sub-processes (irrespective of their granularity level). The *Chemical entity* class contains as subclasses the participants (BioE) of a biological process, e.g. molecules, proteins, molecular complexes, polymers, etc. The *Sequence feature* class contains as subclasses any sequence patterns carried by molecules. Polymers such as DNA and RNA (which belong to the class *Chemical entity*) act as template (have a matrix role): they carried different sequences patterns (e.g. promoter sequences, transcription factor binding site, ribosome binding site, pausing site for ribosomes, etc.). Some of these polymers can participate in several processes. For instance, the same mRNA can be an input of the translation process and of the mRNA degradation process. However, the molecular complexes or proteins involved in these distinct biological processes recognize the sequence patterns. For instance, in Figs. 1 and 2, the specific mRNA sequence patterns named “GGAGG” and “AUG” are involved in two successive elementary processes. Effectively, the inputs of these processes are thus the sequence patterns, and not the whole mRNA itself. When a process is decomposed as in the previous example, we choose to use the sequence patterns of polymers as process participants instead of the molecules themselves. In addition, sequence patterns and molecular complexes have to interact and thus have to share a common localization on chromosomes or mRNAs. We defined the *Cellular component* class, which contains as subclasses the parts of cells in which molecules can be localized, and the polymers that carried sequence patterns or bounded chemical entities. In the case of bacteria (a cell without organelle), *Cellular component* class contains the cytosol and polymers such as chromosome or mRNA.

##### Class hierarchy and subclass property

Inside the four main classes, subclasses are organized according to the *is_a* relation to get a Directed Acyclic Graph (DAG) structured model. Unlike in a tree, a class can not only have several subclasses but also be a subclass of several classes (multiple inheritance). The hierarchy of the classes that were imported from GO, ChEBI and SO is kept within the DAG model. Processes, chemical entities and patterns are placed as leaves of the bioBiPON DAG model.

##### Importation and interoperability

**Fig. 3:**
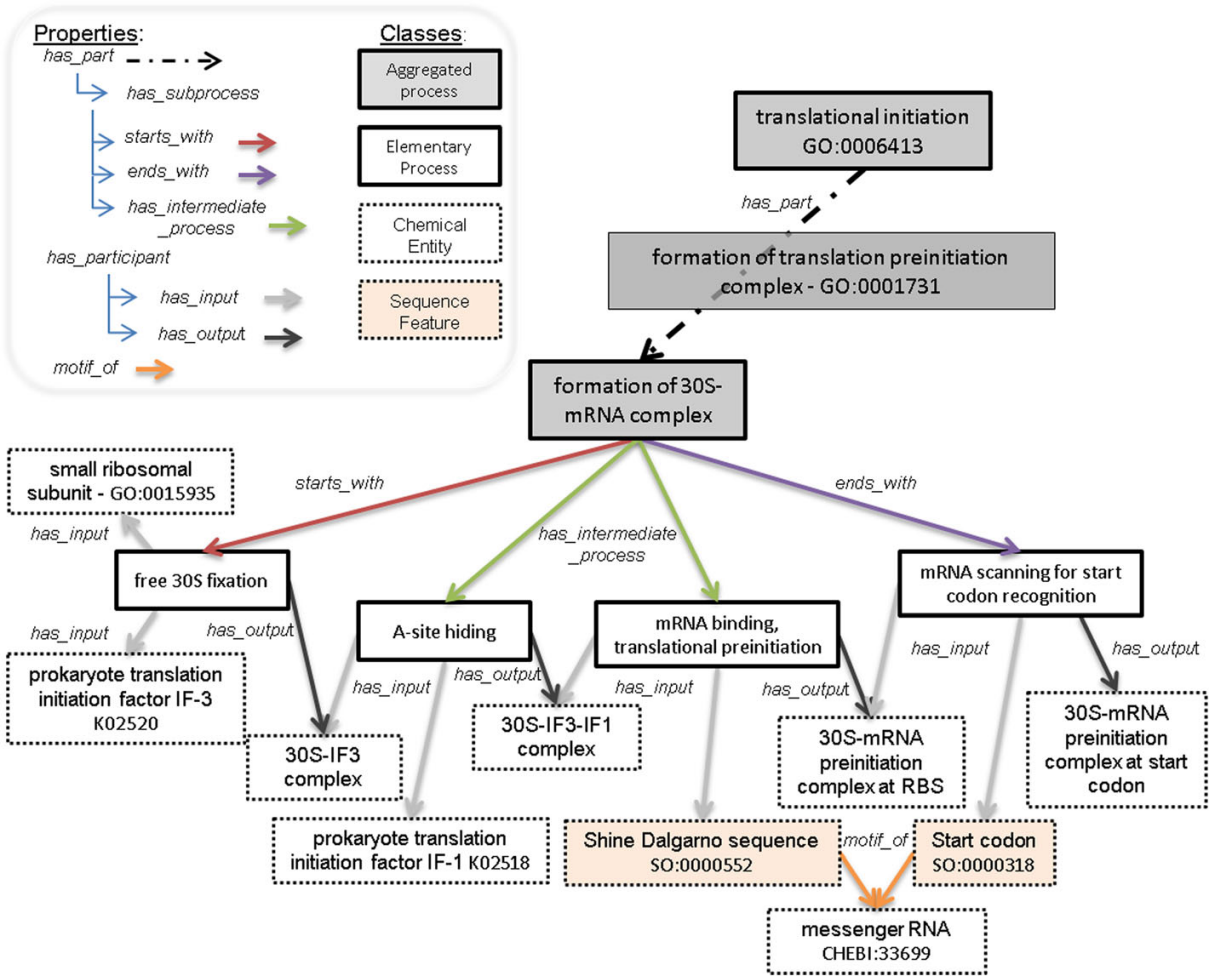
Ontological representation of the formation of 30S–mRNA complex in bioCMON into interlocked subsystems. The aggregated process formation of 30S–mRNA complex is composed of four elementary processes: “free 30S fixation”, “A-site hinding”, “mRNA binding translational preinitiation” and “mRNA scanning for start codon recognition”. Whenever a class was imported from an existing bio-ontology (GO, ChEBI, KEGG or SO), the original Id is indicated

For all classes and properties that were imported from other bio-ontologies (e.g. translation initiation; see Fig. 3), we kept the original references, such as the Internationalized Resource Identifier (IRI) and Identifier (id) in bioBiPON, to ensure interoperability. In BiPON, the SO classes of gene products are now considered as subclasses of the *Chemical entity* class instead of the *Sequence feature* class. Due to this semantic change, we considered these SO classes as new classes: we gave a new IRI and kept the original one with the *hasDbXref* annotation (e.g. hasDbXref SO_0000185). When a class refers to a term in an existing database (such as KO), the original id is also kept with the *hasDbXref* annotation (e.g. prokaryote translation initiation factor IF-3: hasDbXref K02520; see Fig. 3).

##### Labeling

For any imported class, the original label is still used in bioBiPON. For any newly created class, we have manually defined a label that was the most representative of the biological process, molecule or sequence represented by the class. The final label can be (a) a term commonly used in the biological schemes of peer-reviewed articles that we considered, or else (b) a Wikipedia term and, otherwise, (c) a term that we chose by taking into account length, completeness and non-ambiguity criteria.

#### Main properties

Properties were partly imported from the Relation Ontology (RO) [28] and partly created manually. Two main properties, *has_participant* (RO_0000057; (INVERSE OF *participates_in* RO_0000056)) and *has_part* (BFO_0000051) were used to formalize elementary or aggregated processes, respectively. The *has_participant* property includes the sub-properties *has_input* (RO_0002233), *has_output* (RO_0002234), and *has_catalyst*. In the ontological model, they are represented by arrows between the biological processes and the BioEs (see Fig. 3). These properties are used to formalize relations between elementary processes. The *has_part* property is transitive and is further specialized into two intransitive sub-properties called *cyclication_of* and *has_subprocess*. The *has_subprocess* is further specialized into *starts_with*, *ends_with*, *has_intermediate_process* and *has_fork_process* disjoint sub-properties that can be used to formalize aggregated processes. The *has_part* property enables the decomposition of an aggregated process along the granularity levels down to elementary processes, while the *has_subprocess* property manages the relation between two successive granularity levels. *starts_with*, *ends_with*, *has_intermediate_process* and *has_fork_process* participate in the management of successive processes that are part of a process of the same granularity level. The properties *starts_with* and *ends_with* define which sub-process starts and ends the aggregated process respectively. We further define the property *has_fork_process* in the case of several sub-processes start an aggregated process. The properties *has_intermediate_process* define the sub-processes that occur between the starting and the ending sub-processes.

The *located_in* property is used to define the localization of *Chemical entity* class inside the cell.

As mentioned above, the *Chemical entity* and *Sequence feature* classes are in relation through the *is_motif_of*, *binds_to* and *has_template* properties. The transitive property *is_motif_of* localizes the sequence patterns in a larger one and finally in a polymer. The *binds_to* property (a *located_in* sub-property) defines the sequence where a *Chemical entity* binds a polymer. The *has_template* property points out a sequence that affects the recruitment of a specific *Chemical entity*.

##### Formal definition of biological processes

**Fig. 4:**
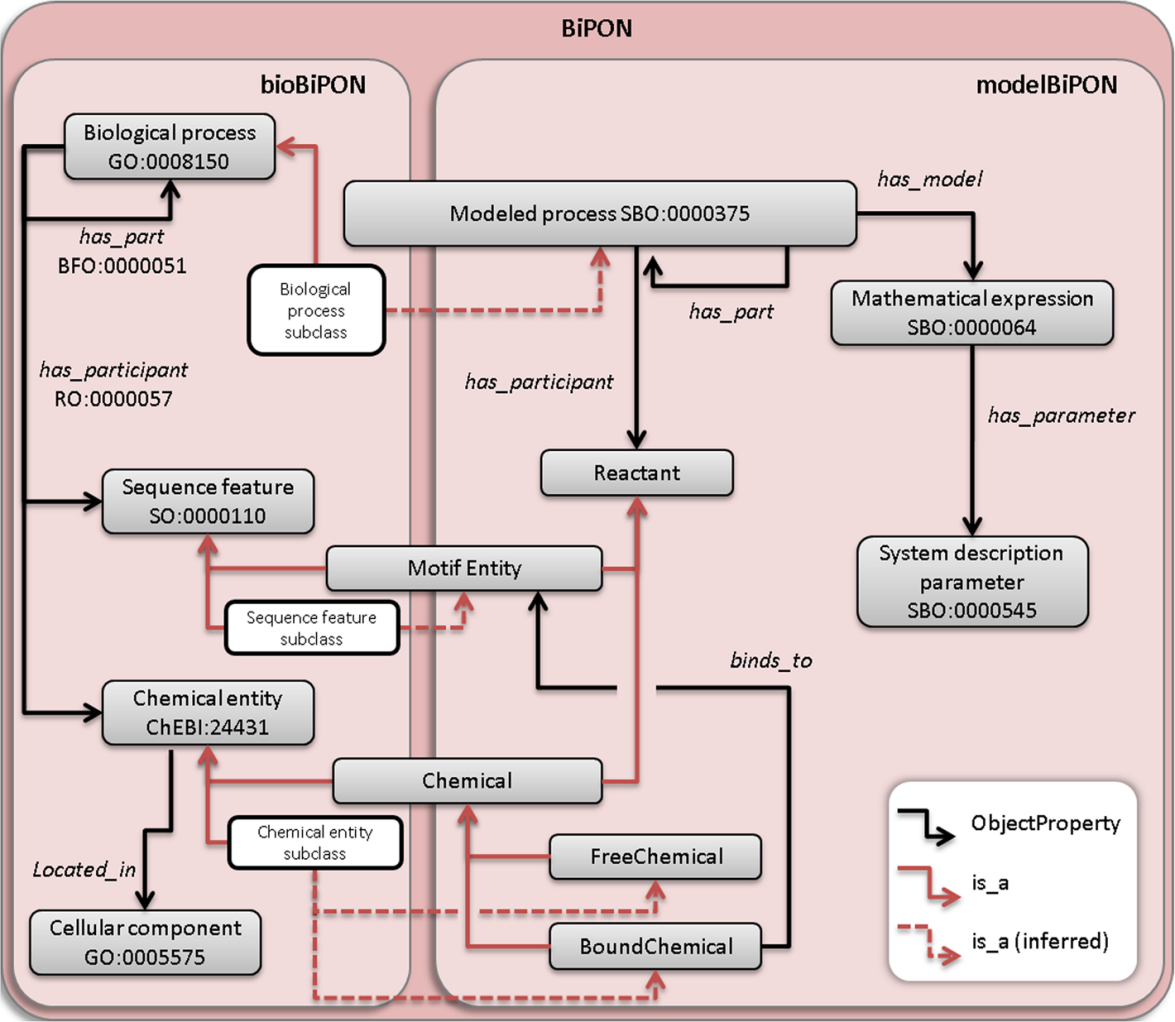
BiPON structure: focus on automatic reasoning on bioBiPON and modelBiPON. Dotted red arrows represent the inferred relation between classes of bioBiPON and modelBiPON using automatic reasoning, while straight red arrows stand for the standard *is_a* property. Abbreviations are GO: Gene Ontology; SO: Sequence Ontology; ChEBI: Chemical Entities of Biological Interest; SBO: System Biology Ontology; BFO: Basic Formal Ontology; RO: Relation Ontology

We used the Protégé editor, which is based on Description Logics, to formalize the classes. We distinguished two kinds of classes, namely primitive classes, which are described by necessary conditions (e.g. subclass of other classes), and complex classes, which are defined by equivalence using both necessary and sufficient conditions. Thus, the formal definition of classes follows templates that may combine universal (ONLY) and existential (SOME) restrictions [27]. The structure of bioBiPON is displayed on Fig. 4.

*Elementary process* class is related to *chemical entity* or *sequence features* classes via h*as_participant* sub-properties by the following general class axiom:

elementary_process ≡ has_input SOME chemical entity AND has_output SOME chemical entity AND has_input ONLY (chemical entity OR sequence feature) AND has_output ONLY (chemical entity OR sequence feature).

In the previous definition, *Chemical entity* is a primitive class (defined as a subclass of bioBiPON), while *elementary_process* is a class defined by equivalence using two kinds of restrictions. Any subclass of *elementary_process* must have at least one *Chemical entity* subclass as an input and as an output. Moreover, the inputs and the outputs of a subclass of *elementary_process* must be either subclasses of *Chemical entity*, either subclasses of *Sequence feature*.

For instance, the *elementary process* subclass *Free 30S fixation* in Fig. 3 is defined as follows:

Free 30S fixation ≡ has_input SOME IF3 AND has_input SOME 30S AND has_output SOME 30S–IF3 complex AND has_input ONLY (IF3 OR 30S) AND has_output ONLY 30S IF3 complex.

In this definition of the class *Free 30S fixation*, we specialized the type of chemical entity that at least one input (output) must satisfy. For instance, one input has to belong to the class *IF3*, a sub-class of *chemical entity*.

*Aggregated process* class are related to *cellular process* class via *has_part* sub-properties according to the following general class axiom:

aggregated_process ≡ has_subprocess SOME cellular process AND has_subprocess ONLY cellular process.

For instance, the *aggregated process* subclass *Formation of 30S–mRNA complex* in Fig. 3 is defined as follows:

Formation of 30S–mRNA complex ≡ starts_with SOME free 30S fixation AND has_intermediate_process SOME A site hiding AND has_intermediate_process SOME mRNA binding, translation preinitiation AND ends_with SOME mRNA scanning for start codon recognition AND has_subprocess ONLY (free 30S fixation OR A site hiding OR mRNA binding, translation preinitiation OR mRNA scanning for start codon recognition).

Fig. 3 illustrates the ontological representation of the formation of 30S–mRNA complex into aggregated and elementary processes using the classes and properties of bioBiPON.

### modelBiPON ontological model

The ontological model called modelBiPON aims at relating generic biological processes to their mathematical models including parameters. Knowledge about mathematical models was gathered from two sources. A first flat source of knowledge was provided by systems biology specialists who established a list of generic, useful and well-established models of biological processes. The second source of knowledge was a selection of ontology classes that were directly imported from SBO, more specifically from the *mathematical expression* (SBO:0000064) and the *system description parameters* (SBO:0000545) classes. These classes and subclasses include fairly enough pieces of knowledge for laws and parameters, respectively.

#### Main classes

We defined four main classes: *Modeled process*, *Reactant*, *Mathematical expression* and S*ystem description parameters* (Fig. 4).

The *Modeled process* class corresponds to the *process* class of SBO (SBO:0000375) and contains, as subclasses, specific biological processes of bioBiPON for which it exists a mathematical model. The *Reactant* class is an abstract representation of the inputs and outputs of a *Modeled process*. *Reactant* is specialized into two disjoint subclasses, *Motif Entity* and *Chemical*, corresponding to the subclasses of *Sequence feature* and *Chemical entity* of bioBiPON that are inputs/outputs of a *Biological process* (see Fig. 4). The *Chemical* class is further specialized into the disjoint subclasses *Free Chemical* and *Bound Chemical*. Subclasses of *Free Chemical* represent the chemical entities that are freely available to interact with any other chemical entities in the cytoplasm. The subclasses of *Bound Chemicals* represent molecular complexes composed of one or several chemical entities that are bound specifically to a sequence pattern. The *Modeled process* and *Reactant* classes are abstract representations and are therefore at the top level of the modelBiPON ontology.

*Mathematical expression* and S*ystem description parameters* include subclasses from SBO and subclasses that were defined according to the mathematical models. For the classes that were imported from SBO, we carefully kept the IRIs and the Ids to ensure interoperability between ontologies.

#### Main properties

In modelBiPON, the sub-properties of *has_participant* in bioBiPON were used to relate the classes of *Modeled process* and *Reactant* while the sub-properties of *has_part* managed the decomposition of *Modeled process*. Two new types of properties were defined (Fig. 4): *has_model* and *has_parameter*. The *has_model* property links the *Modeled process* and *Mathematical expression* classes while the *has_parameters* property links the *Mathematical expression* and S*ystem description parameter* classes (Fig. 4).

#### Formal definition of modeled processes

The *Modeled process sub*classes are defined by the specificity of their participants (belonging to the class *Reactant*) or by the nature of their discriminating sub-processes. For instance, the most common process belonging to *Modeled process* is *elementary chemical process*. By definition, an *elementary chemical process* has exclusively participants in the *Chemical* class:

elementary chemical process ≡ elementary process AND has_input ONLY Chemical AND has_output ONLY Chemical.

In the same way, a *Sequence binding process* corresponds to the elementary process of binding a *FreeChemical* to a *Motif Entity* and leads to the formation of a *BoundChemical*. This process is formalized as follows:

sequence binding process ≡ elementary process AND has_input SOME Motif entity AND has_input SOME FreeChemical AND has_output SOME BoundChemical AND has_input ONLY (Motif entity OR FreeChemical) AND has_output ONLY BoundChemical.

Aggregated processes that are included in *Modeled process* might be defined by the nature of their discriminating sub-processes such as *Matrix dependent process* and *Polymer production process*:

matrix dependent process ≡ aggregated process AND has_part SOME Sequence binding process.

polymer production process ≡ matrix dependent process AND has_part SOME Release process.

In the previous formal definition, *Release process* is also a subclass of *Modeled process*.

Finally, a *Modeled process* can be refined using the biological property of its participants. For example, the *transcription process* and *translation process* are defined in modelBiPON as follows:

Transcription process ≡ native polymer production process AND has_output SOME primary_transcript.

Translation process ≡ native polymer production process AND has_output SOME pre-process polypeptide.

### BiPON consistency with GO, ChEBI, SO and SBO

To evaluate the logical consistency of BiPON with respect to GO, ChEBI, SO and SBO, we imported the whole set of classes of each ontology into BiPON. Then, we ensured logical consistency using the HermiT 1.3.8 reasoner within the Protégé editor [29].

## Results

### Reasoning on bioBiPON using SWRL rules

Since the BiPON ontology is described using DL syntax, automatic reasoning can be performed within the DL SROIQ framework [29]. However, reasoning on classes alone has its limitations, especially when negations or properties intersection need to be handled [30]. This difficulty can be bypassed by instantiation of classes. We first instantiated leaf-classes with a unique and distinct individual *indiv*: *i_NameOfTheClass*, *s_NameOfTheClass*, and *p_NameOfTheClass* for the *Chemical entity*, *Sequence feature* and *Biological process* leaf-classes respectively. Leaf-classes were restricted to these singletons. We assume that the unique individual *indiv* is considered as the typical member of its class. Other individuals can be instances of that class, but they will all be inferred to be *the same as* the typical individual *indiv*, since each singleton leaf-class is defined as *equivalent to* {indiv}. After the instanciation, we designed rules in Semantic Web Rule Language (SWRL), as supported in Protégé, to formalize additional constraints between classes and properties [30].

#### Automatic input/output building of aggregated processes

In the rest of the paper, we call “sub-process of a process *p* “any process that is related to *p* by a *has_subprocess* property.

**Fig. 5:**
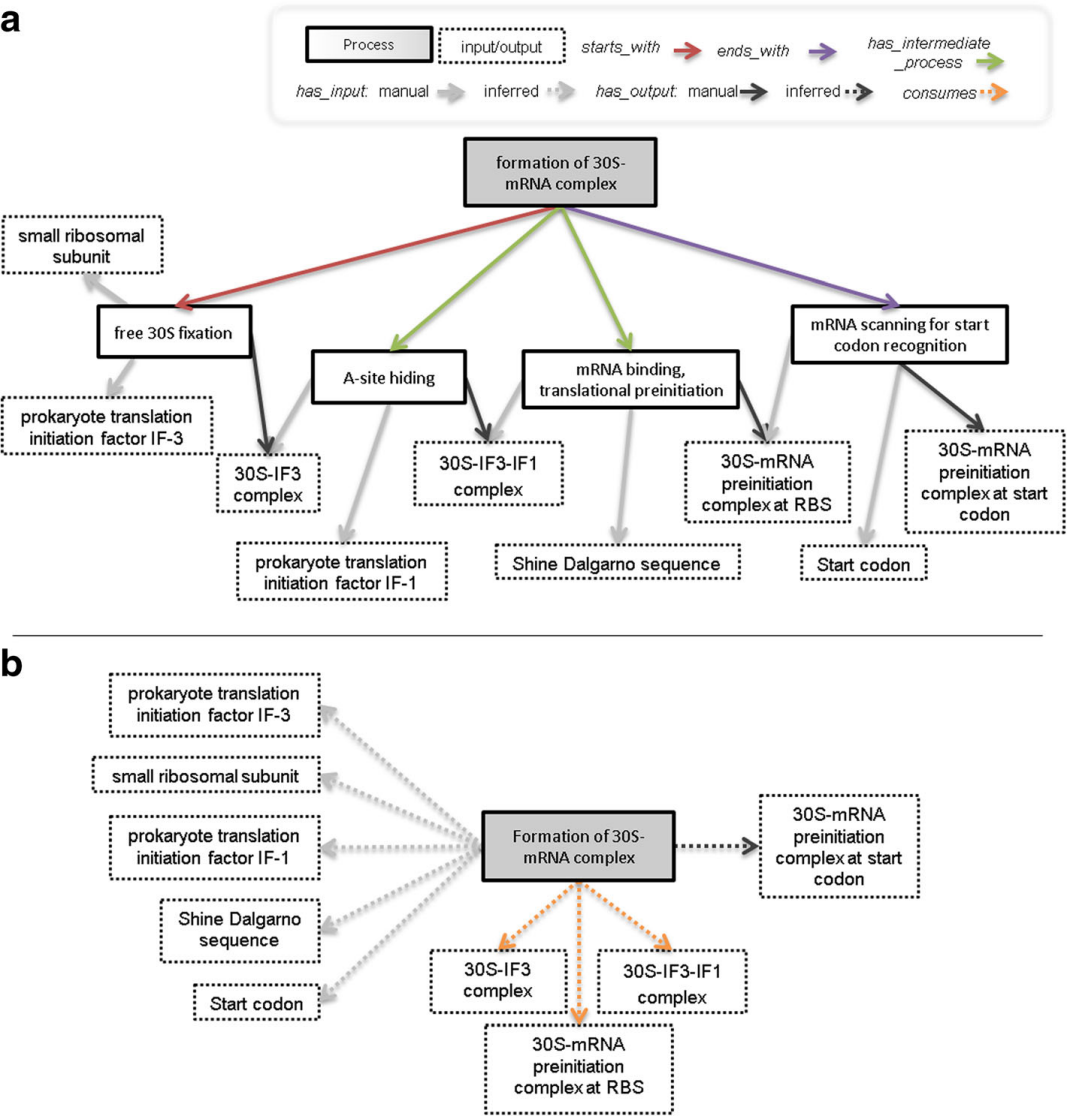
Inputs/outputs aggregation for the formation of 30S–mRNA complex. **a** Formal description of formation of the 30S–mRNA complex and manual *has_input* and *has_output* properties of elementary processes. **b** Inferred *has_input*, *has_output* and *consumes* properties that were designed using SWRL rules after HermiT automatic reasoning

Elementary processes are manually defined by specifying the classes of their inputs and outputs using *has_input* and *has_output* properties, while aggregated processes are manually defined by the composition of their sub-processes (see Fig. 5a). The naive composition of *has_participant* (*has_input* or *has_output*) and *has_subprocess* sub-properties would result in the list of all inputs/outputs of elementary processes. However, intermediate macromolecules that are produced and consumed by two successive elementary processes should be removed from the inputs/outputs of aggregated processes (see Fig. 5b).

To overcome this difficulty, we built the inputs/outputs of aggregated processes by automatic reasoning using SWRL rules in two steps.

- *Step1:* We identified a first set of input(s) and output(s) of the aggregated process: (a) the input(s) of all sub-processes that start the aggregated process and (b) the output(s) of the sub-process that ends the aggregated process. In formal language, we have:
  - Let p_agg be an aggregated process. IF pagg starts with p_sub and p_sub has the molecule chem_i as an input, THEN p_agg has the molecule chem_i as an input. And IF pagg has as fork process p_sub and p_sub has the molecule chem_i as an input, THEN p_agg has the molecule chem_i as an input. And IF pagg ends with p_sub and p_sub has the molecule chem_i as an output, THEN p_agg has the molecule chem_i as an output.
- *Step2:* We identified a second set of inputs and outputs of the aggregated process, containing any input or output that is not produced then consumed by two successive intermediate sub-processes. To do so, we first had to determine the successive order of two sub-processes in an aggregated process. We defined the property *precedes* as follows. Since by definition two successive elementary processes are linked by one intermediate molecule, the process that provides the molecule (as output) *precedes* the process that consumes the molecule (as input).
  Let p_agg be an aggregated process and p_sub1 and p_sub2 be some distinct processes. IF p_sub1 and p_sub2 are sub-processes of p_agg and p_sub1 has the molecule chem_i as an output and p_sub2 has the molecule chem_i as an input, THEN p_sub1 precedes p_sub2.

Then any output of the first sub-process that is not an input of the second sub-process, will be an output of the aggregated process. Conversely, any input of the second sub-process that is not an output of the first sub-process will be an input of the aggregated process.

Let p_agg be an aggregated process that starts with a sub-process, let p_sub1 and p_sub2 be some successive processes, and let chem_i and chem_j be different molecules. IF p_sub1 has the molecule chem_i as an output and p_sub2 has the molecule chem_i as an input and p_sub2 has the molecule chem_j as an input, THEN p_agg has the molecule chem_j as an input.

#### Automatic identification of consumed participants

We also identified intermediate molecules that are consumed by an aggregated process, e.g. molecules that are produced and then consumed by two successive elementary processes.

IF an aggregated process p_agg is composed of sub-processes p1 and p2. IF p_agg starts with process p_starts and ends with process p_ends, IF the macromolecule m is an output of p1 and the macromolecule m is an input of p2 and p1 is different from p2, IF p1 is different from p_ends, and IF p2 is different from p_starts, THEN p_agg consumes m.

An example, the aggregation of inputs and outputs of the “formation of 30S–mRNA complex” process is given on Fig. 5. The process is represented twice, before (Fig. 5a) and after (Fig. 5b) aggregation. In Fig. 5b, we highlight the three intermediate molecules (30S–IF3 complex, 30S–IF3-IF1 complex and 30S–mRNA preinitiation complex at RBS) that were identified with the *consumes* property. These three intermediate molecules do not appear as participants in the aggregated process.

#### Automatic identification of key participants

For some elementary processes, an input participant can also appears as an output participant. Such a participant is a reactant that is necessary to the realization of the process, but that is not modified in the process. This participant can then be considered as a key component of the elementary process. An example of such a participant is the enzyme that catalyzes an enzymatic reaction. Identification of the key participants of aggregated processes is obtained after automatic aggregation of inputs and outputs. To identify these key participants by automatic reasoning, we designed the new property *has_key_element* with an SWRL rule (see Additional file 2 for the formal definition):

IF m is a macromolecule and p has m both as an input and as an output THEN p has m as a key element.

The key elements of a process can further be linked to this process by means of the inverse property of *has_key_element*: *key_element_of*. A macromolecule may be a key element of several different processes, which in turn may be involved in different cellular functions. The property *key_element_of* enables to find all processes for which a macromolecule appears as a key element. For instance, the “Class I translation release factor” (RF, BiPON_00000361) is *key_element_of* the processes “ArfA system rescue” (BiPON_00001193) and “bacterial cytoplasmic translational termination” (BiPON_00002268). By rating this statement, we automatically point out the dual functional role of RF in the translation process. In total, we obtained 44 key elements for the processes contain in the bacterial gene expression.

### Reasoning on bioBiPON and modelBiPON

To establish relationships between the sub-ontologies bioBiPON and modelBiPON, we applied automatic inference. We first defined the *Reactant* class and subclasses of modelBiPON by bridge rules with bioBiPON subclasses. Inference then proceeds in two steps based on the formal definition of modelBiPON (Fig. 4 red dotted arrow): (1) a linking operation between *Chemical entity* or *Sequence feature* of bioBiPON and *Reactant* of modelBiPON, and then (2) a hierarchical classification of *Biological process* of bioBiPON within *Modeled process* of modelBiPON according to their model and participant characteristics obtained in step 1 through the *is_a* property. *Modeled process* and *Reactant* classes from modelBiPON are then filled with a set of *Biological process* and *Chemical entity* subclasses from bioBiPON, respectively. This consists in an automatic selection of biological processes for which a mathematical model exists (Fig. 4 dotted red arrow).

#### Definition of bridge rules

For the first step of automatic inference, bridge rules between the classes of both ontologies must be defined. The *Reactant*, *Chemical*, and *Motif Entity* classes as well as their subclasses in modelBiPON are related, via these rules, to the *Chemical entity* and *Sequence feature* classes in bioBiPON. More precisely, the class *Reactant* is defined as follows:

Reactant ≡ participates_in SOME biological process.

As stated in the Methods section, the *Chemical* and *Motif Entity* subclasses of *Reactant* are disjoint. The *Chemical* class includes subclasses of *Chemical entity*, while subclasses of *Motif Entity* in modelBiPON correspond to subclasses of *Sequence feature* in bioBiPON:

Chemical ≡ Reactant AND Chemical entity.

Motif Entity ≡ Reactant AND Sequence feature.

Moreover, due to their importance for modeling purposes, we formally defined the *Free Chemical* and *Bound Chemical* subclasses as follows:

FreeChemical ≡ Chemical AND located_in ONLY cytosol.

BoundChemical ≡ Chemical AND binds_to SOME Sequence feature.

Since *binds_to* is a sub-property of *located_in*, the localization of *Bound Chemical* corresponds to the one of *Motif entity*.

#### modelBiPON filling by automatic reasoning

**Fig. 6:**
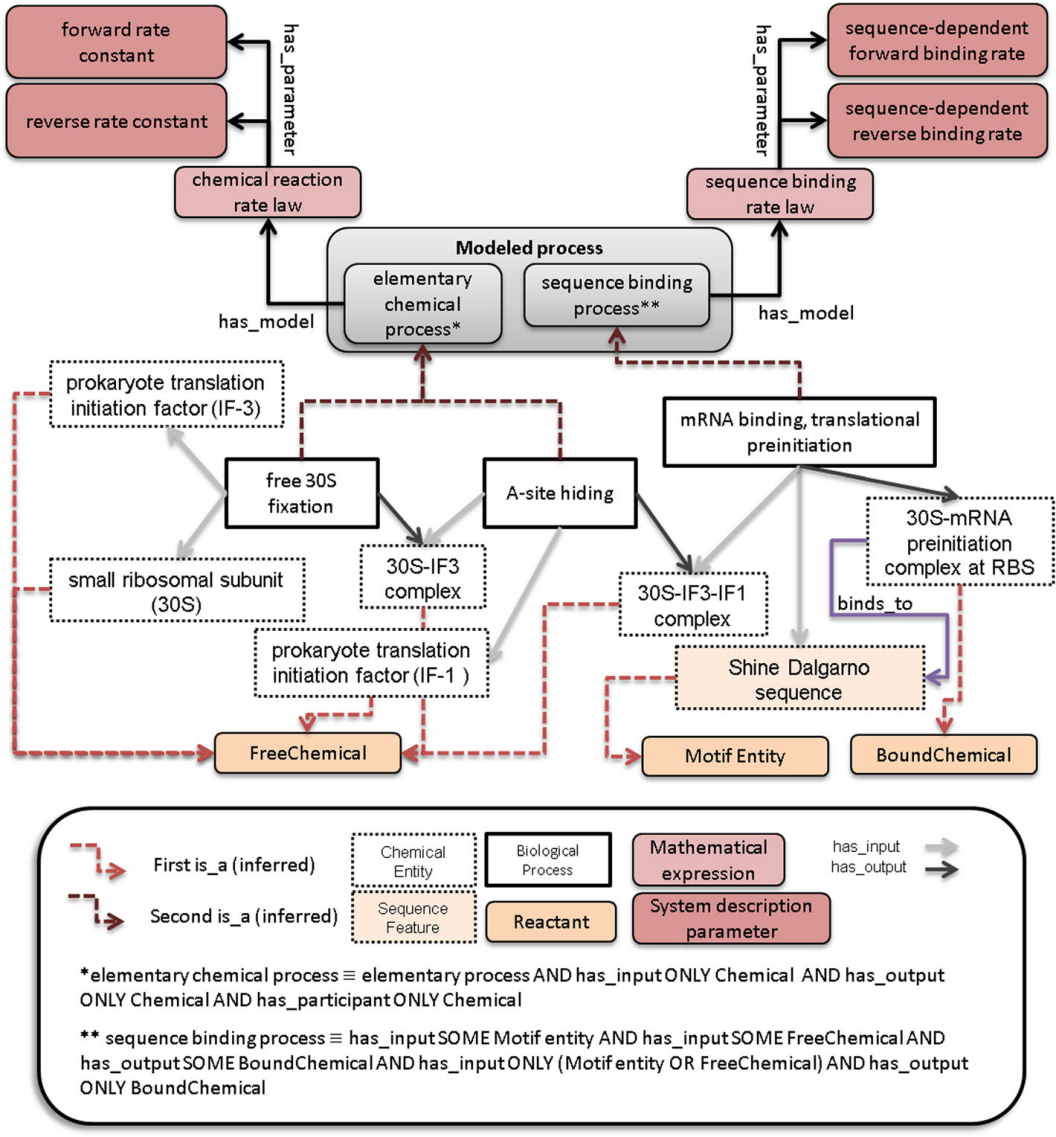
Results of reasoning on bioBiPON and modelBiPON for elementary processes involved in the formation of the 30S–mRNA complex

The class hierarchy of the *Reactant*, *Chemical*, and *Motif Entity* classes was inferred by automatic reasoning. Once initial inputs and outputs of a *Biological process* class in bioBiPON are related to the abstract class *Reactant* of modelBiPON, the *Modeled process* class hierarchy is performed by automatic reasoning. At last, the most specialized subclasses for the *is_a* relation of the *Reactant* and *Modeled process* classes in modelBiPON are the leaf-classes of bioBiPON. Fig. 6 illustrates the results of the inference process for an example, the formation of the 30S–mRNA complex.

### Computing performance

Using HermiT 1.3.8 [29] within the Protégé editor, the consistency of BiPON can be computed in less than 5 s. The automatic building of the class hierarchy (including the identification of inputs/outputs of aggregated processes and of key participants) and the inference of relationships between bioBiPON and modelBiPON takes 240 min (2.33GHz, 16Go).

### Flexibility and genericity

New aggregated processes can be easily defined by specifying (a) the elementary sub-processes to be aggregated using the *has_subprocess* sub-properties. The inputs/outputs of the aggregated process and the consumed molecules can then be inferred by automatic reasoning. In addition, participant element that have a key role in elementary or aggregated processes can be automatically determined. Altogether, combining DL syntax, SWRL rules and automatic reasoning make BiPON highly flexible and generic regarding the addition of new processes, participants, or models. Due to the flexibility of SWRL, new rules can be created and added easily. For test purposes, we provided a simple ontological model (toyBiPON) that is representative of BiPON in Additional file 3. This model is schematically described in Additional file 4.

### BiPON content

The set of BiPON statistical metrics and mapping is presented on Table 1. The ontology BiPON consists of 1746 classes (including 767 distinct individuals representing leaf-classes) and 30 object properties. Definitions of classes use 15,054 Axioms, including 4265 logical axioms, 8 SWRL rules for identification of the inputs/outputs of aggregated processes and 2 SWRL rules for alternative biological information feature (identification of key participants and consumed molecules).

**Table 1:** Numbers and provenance of BiPON classes

	Numbers		Provenance of classes (nb of classes)				
Class name	Class count	% of BiPON	GO	ChEBI	KEGG	SO	SBO
BiPON	1746	100,0%	145	183	186	92	49
└ modelBiPON	194	11%	–	–	–	–	49
└ bioBiPON	1582	91%	145	183	186	92	–
└ biological process	551	32%	123	–	34	–	–
└ elementary process	243	14%	29	–	32	–	–
└ aggregated process	131	7%	21	–	–	–	–
└ Cellular component	19	1%	5	1	–	5	–
└ Chemical entity	754	43%	17	183	152	41	–
└ Sequence feature	234	13%	–	–	–	51	–
input/output (i/o)^a^	514	29%	8	90	144	36	–
└ i/o AND molecule	34	2%	–	34	–	–	–
└ i/o AND macromolecule	444	25%	8	56	144	28	–
└ i/o AND sequence feature	36	2%	–	–	–	8	–

The proportions in the columns “Numbers” are computed with respect to the total class count (1746) of BiPON

^a^ ≡ Reactant

bioBiPON classes cover 91% of BiPON classes. One third of them are subclasses of *Biological process*, and two thirds are *Chemical entity* or *Sequence feature* subclasses. The difference in proportion is due to the fact that a *Biological process* is defined by different participants in input-output. 28% of the *Biological process* classes were imported from GO (123) and KEGG (34), while 45% of the participants were imported from GO (17), ChEBI (183), SO (92) and KEGG (152; Table 1). Classes imported from GO and SO were mainly used for hierarchy building, classes imported from ChEBI and especially KEGG were mostly used as leaf-classes. The remaining 976 non-imported classes (62%) were manually designed. modelBiPON classes represent only 11% of BiPON classes due to their abstract representation. This low coverage of BiPON classes by modelBiPON classes is particularly interesting. Despite their apparent diversity, many biological processes can thus be represented by the same type of mathematical models. Most of modelBiPON classes are distributed between *mathematical expression* and *system description parameters* and 26% of them were imported from SBO (49).

*Biological processes* include 243 elementary processes that are successively aggregated in 131 aggregated processes up to final aggregates. The elementary processes have 514 participants as input or output: 34 of this participants describes molecules, 444 gene products or molecular complex, 36 sequences. Altogether, molecules and sequence patterns represent only 14% of the inputs or outputs of biological processes, but they are involved in the definition of 63% of elementary processes. These proportions highlight the difference between basic molecules (e.g. water, ions or ATP) and sequence patterns (e.g. codons), which are often involved in many biological processes of gene expression, and macromolecules such as proteins, which are usually specific to a few biological processes.

After automatic reasoning, *Modeled process* classes include 213 elementary processes subclasses and 66 aggregated processes subclasses of bioBiPON. A large proportion among them (69%) are *Chemical process* subclasses in modelBiPON and was automatically linked to the mathematical expression “*chemical reaction rate law”* and its parameters (see Fig. 5). The remaining 31% correspond to other types of mathematical models, such as “*sequence binding rate law*” for *Sequence binding process*.

Currently, bioBiPON contains 77 biological processes subclasses that have no associated mathematical model and are therefore not included in modelBiPON. These classes correspond to intermediate processes that are not critical in a modeling perspective, but interesting for the biological knowledge description. However, in the future, if a mathematical model was built for them, it could be included in modelBiPON straightforwardly.

## Discussion

In engineering science, the development of complex systems such as airplanes or nuclear power plants involves the development of specifications to ensure that the whole system will function in normal and/or degraded modes. As a prerequisite, such specifications will usually contain a catalog of sparse parts and an interaction map of the entire system. Each piece of the system must be described and characterized. To tackle the intrinsic complexity of managing thousands of entities, the design of the whole system is achieved by using a systemic approach: the whole system is broken down into sub-systems of lower complexity, where each sub-system is well characterized and can be simulated. Adopting such a systemic representation was clearly the cornerstone of the development of complex engineering systems [11]. At the beginning of the twenty-first century, the field of systems biology was established based on exactly the same idea: that a cell is a complex system and that a systemic representation can help understand how the whole cell works [10]. Kitano’s analysis in [10] appeared to be especially fruitful. Since 2012, the first developed whole-cell models were based on the systemic description of cells and showed a high capability of prediction [1, 2]. This effectively demonstrates the relevance of the systemic approach to progress in the understanding of living organisms. Systems biology can thus greatly profit from systematically importing relevant concepts and know-how from engineering science into the biological field [10].

In this article, we introduced the ontology BiPON, which is intrinsically based on a systemic representation of cells [1, 6, 7]. In particular, BiPON focuses and clarifies the notion of biological processes by breaking down the cell into subsystems and by automatically relating them to mathematical models. Each subsystem is formally defined in terms of the cellular components it contains. Consequently, the function of each cellular component is conditioned to the subsystem to which it belongs. If a cellular component participates in multiple subsystems, it can also have different functions in the cell. In other words, the function of a cellular component now depends on the biological processes in which it is involved, and not only on its own chemical properties.

In describing biological knowledge, BiPON relies as much as possible on existing, well-established and commonly used bio-ontologies (GO, ChEBI, SO, SBO) in order to avoid the conception of unnecessary classes and, thus, to prevent redundancy. To ensure interoperability, we carefully stored IRI or Ids of each imported class and added new classes only when necessary. Approximately 72% of the *Biological process* classes in BiPON were created manually (see Table 1). Among the new *Biological process* classes, we created 175 elementary processes to further refine the biological description of bacterial cellular processes, thereby contributing to enrich the biological knowledge for prokaryotes. Finally, 78% of the *Biological process* classes in BiPON were linked to mathematical models by automatic reasoning. Altogether, combining the systemic description of the cell with an ontology enabled us to detect and fill gaps in the description of bacterial gene expression. BiPON is suited to help users to refer and share the same concepts regardless of their scientific background (biologist, mathematician, etc).

BiPON complements several ongoing efforts of the GO consortium, including GO-plus [31–33] and the Linked Expression using the Gene Ontology (GOCAM) formalism [32], which aim at promoting the comprehension, consistency and integration of biological knowledge within ontologies. GO-Plus provides relations between classes in GO-BP, GO-CC, GO-MF, metabolite classes in ChEBI, and polymer classes in SO. Compared to GO-plus, BiPON went a step further by integrating the SO sequence patterns involved in bacterial gene expression, and the mathematical models and related parameters of SBO. Specific sequence patterns had to be integrated because they are involved in 33% of elementary processes as participants. Beyond their content, GO-Plus, GOCAM and now BiPON provide promising formal frameworks to relate biological processes to their molecular functions. In [34, 35], the authors suggested that the formal relationship between GO-BP and GO-MF should be refined to improve the representation of biological knowledge. GO-Plus includes GO-BP and GO-MF sub-ontologies [33] without the addition of new relations. Within the GOCAM project [32], the GO consortium has defined a new relation (*affects*) to link GO-MF to GO-BP. Using BiPON, we expect to infer such links by automatic reasoning. For example, we automatically identified in BiPON the key participant of a biological process as the relevant *Chemical entity* that is necessary and unchanged during a *Biological process*. In the case of an enzymatic reaction, the key participant is the enzyme itself. Relating biological processes to molecular functions in BiPON could be achieved as follows: IF a chemical entity is a “key element” of a “process” and “has_function” a “molecular function”, THEN this “molecular function” “affects” the “process” and all “aggregated process” of higher level (see Additional file 2 for the formal SWRL rule). We provided an example of such a reasoning in the toyBiPON ontology (see Additional file 3). Altogether, this illustrates the usefulness of bio-ontologies through the ability of inferring new relations by automatic reasoning.

Beyond that, ontologies are also widely used to organize data warehouses [36–38]. BiPON is currently used to drive the development of a data warehouse for the bacterial gene expression processes. BiPON supports the design of the relational model of the database that stores and connects biological knowledge, heterogeneous multi-omics data [2, 25, 39, 40], static data such as sequence patterns, and mathematical models [23, 24]. High-throughput technologies already enable the acquisition of data at a large scale: transcriptome, proteome (including post-translational modifications), fluxome, interactome, metabolome, degradome, etc. [41]. In the future, new information could be obtained by combining omics data acquisition with statistical tools and computational algorithms for data analyses [42], model simulations, and accumulated biological knowledge. Typically, the combination of omics data and data analysis methods [43] may help to identify large sets of molecular compounds together with their biological functions, their interactions [44], and some of their kinetic properties, including for instance equilibrium constants or the half-lives of proteins or mRNAs. Using BiPON, any new omics data, new in silico predictions or new dynamical parameter sets (such as half-life of a protein, affinity constant, kinetic parameters, etc.) can be automatically anchored to systemic description of the cell and be linked to appropriate chemical entities or biological process of interest at any scale. BiPON and the data warehouse could serve as a point of entry into a shared resource of information that may be useful for biologists, computational biologists, statisticians and modelers.

BiPON currently contains a rather exhaustive description of the bacterial gene expression (including mechanisms of regulation), i.e. transcription, RNA processing and decay, ribosome biogenesis, tRNA aminoacylation and translation. However we are aware that BiPON is not yet complete and that new classes will have to be added in the future. In fact, the description methodology proposed in this article is highly flexible and generic. We expect that any new process, new participant, or other knowledge resource can be inserted in BiPON and be linked to mathematical models. BiPON is an ongoing project and future releases of BiPON will cover not only other bacterial processes such as DNA replication, cell wall synthesis or metabolism, but also cellular compartments (cytosol, membrane, periplasm, etc.). This will be a key step for extending BiPON to compartmentalized eukaryotic cells.

## Conclusions

In this manuscript, we developed BiPON an ontology dedicated to the systemic representation of bacterial biological processes. This ontology is a proof of concept in several ways. It demonstrates that a large set of interlocked bacterial processes can be formally described, with an ontology, as input/output subsystems on different levels of granularity using a few set of properties and can automatically be linked through inference with their mathematical models and related parameters. The proposed methodology to build the systemic representation of bacterial processes is generic and could thus be easily implemented for other processes. BiPON links elementary entities such as single molecules or sequence patterns to biological processes and enables users to navigate from elementary to high-level processes and vice versa. Finally, combining instantiation and SWRL rules during automatic reasoning on BiPON enrich the knowledge by providing new properties with high flexibility. By interlacing biological knowledge with mathematical models, BiPON should open up promising perspectives for biologists, for data scientists, for computational, and system biologists and more largely for the emerging multi-disciplinary community of researchers studying whole-cell integration, modeling and simulation.

## Additional files

Additional file 1: The list of the main references used to review the bacterial gene expression processes. (PDF 70 kb)

Additional file 2: Table of SWRL rules for the properties *consumes*, *has_input*, *has_output*, *has_key_element*, *affects*, *precedes*, *before*. (TIFF 90 kb)

Additional file 3: A small ontological model that illustrates the usage of BiPON. (OWL 199 kb)

Additional file 4: Graphical representation of the biological processes, chemical entities, and properties included in toyBiPON. (PNG 205 kb)

## Abbreviations

30S: Prokaryote ribosomal small subunit
BioE: Biological entities
BiPON: Bacterial interlocked Process ONtology
cBioE: consumed BioE
ChEBI: Chemical Entities of Biological Interest
DAG: Directed Acyclic Graph
DL: Description Logic
fBioE: final BioE
GO: Gene Ontology
GO-BP: GO biological process
GO-CC: GO Cellular Component
GO-MF: GO Molecular Function
iBioE: initial BioE
IF3: Translation initiation factor
KEGG: Kyoto Encyclopedia of Genes and Genomes
mRNA: messenger RNA
ncBioE: new consumed BioE
OWL: Web Ontology Language
RF: Release Factor protein
RNA: RiboNucleic Acid
RO: Relation Ontology
SBO: System Biology Ontology
SO: Sequence Ontology
SWRL: Semantic Web Rule Language
