# Supplementary material for: The bacterial interlocked process ONtology (BiPON): a systemic multi-scale unified representation of biological processes in prokaryotes
Source: J Biomed Semantics. 2017 Nov 23;8:53. doi: 10.1186/s13326-017-0165-6 (PMC5701433; doi:10.1186/s13326-017-0165-6)
Supplement: Supplementary file 1 — The list of the main references used to review the bacterial gene expression processes. (PDF 70 kb) [file 13326_2017_165_MOESM1_ESM.pdf]

This document provides the list of the main references used to review the bacterial gene expression processes.

References

[1] Qutaiba O Ababneh and Jennifer K Herman. Rela inhibits bacillus subtilis motility and chaining. *Journal of bacteriology*, 197(1):128–137, 2015.

[2] John Achenbach and Knud H Nierhaus. The mechanics of ribosomal translocation. *Biochimie*, 114:80–89, 2015.

[3] David Achila, Megha Gulati, Nikhil Jain, and Robert A Britton. Biochemical characterization of ribosome assembly gtpase rbga in bacillus subtilis. *Journal of Biological Chemistry*, 287(11):8417–8423, 2012.

[4] Xabier Agirrezabala and Joachim Frank. From dna to proteins via the ribosome: structural insights into the workings of the translation machinery. *Human genomics*, 4(4):226, 2010.

[5] Ayman Antoun, Michael Y Pavlov, Martin Lovmar, and Måns Ehrenberg. How initiation factors tune the rate of initiation of protein synthesis in bacteria. *The EMBO journal*, 25(11):2539–2550, 2006.

[6] Cecília M Arraiano, José M Andrade, Susana Domingues, Inês B Guinote, Michal Malecki, Rute G Matos, Ricardo N Moreira, Vânia Pobre, Filipa P Reis, Margarida Saramago, et al. The critical role of rna processing and degradation in the control of gene expression. *FEMS microbiology reviews*, 34(5):883–923, 2010.

[7] Irina Artsimovitch and Robert Landick. Pausing by bacterial rna polymerase is mediated by mechanistically distinct classes of signals. *Proceedings of the National Academy of Sciences*, 97(13):7090–7095, 2000.

[8] Benedikt M Beckmann, Philipp G Hoch, Manja Marz, Dagmar K Willkomm, Margarita Salas, and Roland K Hartmann. A prna-induced structural rearrangement triggers 6s-1 rna release from rna polymerase in bacillus subtilis. *The EMBO journal*, 31(7):1727–1738, 2012.

[9] Ronald R Breaker. Riboswitches and the rna world. *Cold Spring Harbor perspectives in biology*, 4(2):a003566, 2012.

[10] Robert A Britton. Role of gtpases in bacterial ribosome assembly. *Annual review of microbiology*, 63:155–176, 2009.

[11] JD Brown and MD Ryan. Recoding: expansion of decoding rules enriches gene expression. 2010.

[12] Douglas F Browning and Stephen JW Busby. The regulation of bacterial transcription initiation. *Nature reviews. Microbiology*, 2(1):57, 2004.

[13] Guangnan Chen and Charles Yanofsky. Tandem transcription and translation regulatory sensing of uncharged tryptophan trna. *Science*, 301(5630):211–213, 2003.

[14] Roberto Chulluncuy, Carlos Espiche, Jose Alberto Nakamoto, Attilio Fabbretti, and Pohl Milón. Conformational response of 30s-bound if3 to a-site binders streptomycin and kanamycin. *Antibiotics*, 5(4):38, 2016.

[15] Sruti DebRoy, Margo Gebbie, Arati Ramesh, Jonathan R Goodson, Melissa R Cruz, Ambro van Hoof, Wade C Winkler, and Danielle A Garsin. A riboswitch-containing srna controls gene expression by sequestration of a response regulator. *Science*, 345(6199):937–940, 2014.

[16] Margaret M Elvekrog and Ruben L Gonzalez Jr. Conformational selection of translation initiation factor 3 signals proper substrate selection. *Nature structural & molecular biology*, 20(5):628–633, 2013.

[17] Vitaly Epshtein, Dipak Dutta, Joseph Wade, and Evgeny Nudler. An allosteric mechanism of rho-dependent transcription termination. *Nature*, 463(7278):245, 2010.

[18] Elena Evguenieva-Hackenberg and Gabriele Klug. New aspects of rna processing in prokaryotes. *Current opinion in microbiology*, 14(5):587–592, 2011.

[19] Tamas Gaal, Michael S Bartlett, Wilma Ross, Charles L Turnbough, and Richard L Gourse. Transcription regulation by initiating ntp concentration: rrna synthesis in bacteria. *Science*, 278(5346):2092–2097, 1997.

[20] Anthony O Gaca, Cristina Colomer-Winter, and José A Lemos. Many means to a common end: the intricacies of (p) ppgpp metabolism and its control of bacterial homeostasis. *Journal of bacteriology*, 197(7):1146–1156, 2015.

[21] Emmanuel Giudice and Reynald Gillet. The task force that rescues stalled ribosomes in bacteria. *Trends in biochemical sciences*, 38(8):403–411, 2013.

[22] Seth R Goldman, Richard H Ebright, and Bryce E Nickels. Direct detection of abortive rna transcripts in vivo. *Science*, 324(5929):927–928, 2009.

[23] Paul Gollnick, Paul Babitzke, Alfred Antson, and Charles Yanofsky. Complexity in regulation of tryptophan biosynthesis in bacillus subtilis. *Annu. Rev. Genet.*, 39:47–68, 2005.

[24] Sandra J Greive and Peter H Von Hippel. Thinking quantitatively about transcriptional regulation. *Nature reviews. Molecular cell biology*, 6(3):221, 2005.

[25] Jonas Gripenland, Sakura Netterling, Edmund Loh, Teresa Tiensuu, Alejandro Toledo-Arana, and Jörgen Johansson. Rnas: regulators of bacterial virulence. *Nature reviews. Microbiology*, 8(12):857, 2010.

[26] Qiang Guo, Yi Yuan, Yanji Xu, Boya Feng, Liang Liu, Kai Chen, Ming Sun, Zhixiu Yang, Jianlin Lei, and Ning Gao. Structural basis for the function of a small gtpase rsga on the 30s ribosomal subunit maturation revealed by cryoelectron microscopy. *Proceedings of the National Academy of Sciences*, 108(32):13100–13105, 2011.

[27] Ivan Gusarov and Evgeny Nudler. The mechanism of intrinsic transcription termination. *Molecular cell*, 3(4):495–504, 1999.

[28] David Hasenöhrl, Robert Konrat, and Udo Bläsi. Identification of an rnase j ortholog in sulfolobus solfataricus: implications for 5'-to-3' directional decay and 5'-end protection of mrna in crenarchaeota. *RNA*, 17(1):99–107, 2011.

[29] Tina M Henkin. Riboswitch rnas: using rna to sense cellular metabolism. *Genes & development*, 22(24):3383–3390, 2008.

[30] Kristina M Herbert, William J Greenleaf, and Steven M Block. Single-molecule studies of rna polymerase: motoring along. *Annu. Rev. Biochem.*, 77:149–176, 2008.

[31] Go Hirokawa, Natalia Demeshkina, Nobuhiro Iwakura, Hideko Kaji, and Akira Kaji. The ribosome-recycling step: consensus or controversy? *Trends in biochemical sciences*, 31(3):143–149, 2006.

[32] Ya-Ming Hou. Cca addition to trna: implications for trna quality control. *IUBMB life*, 62(4):251–260, 2010.

[33] Michael Ibba and Dieter Söll. Quality control mechanisms during translation. *Science*, 286(5446):1893–1897, 1999.

[34] Irnov Irnov, Cynthia M Sharma, Jörg Vogel, and Wade C Winkler. Identification of regulatory rnas in bacillus subtilis. *Nucleic acids research*, 38(19):6637–6651, 2010.

[35] Usheer Kanjee, Koji Ogata, and Walid A Houry. Direct binding targets of the stringent response alarmone (p) ppgpp. *Molecular microbiology*, 85(6):1029–1043, 2012.

[36] Kenneth C Keiler. Mechanisms of ribosome rescue in bacteria. *Nature Reviews. Microbiology*, 13(5):285, 2015.

[37] Lev L Kisselev and Richard H Buckingham. Translational termination comes of age. *Trends in biochemical sciences*, 25(11):561–566, 2000.

[38] Edda Klipp, Wolfram Liebermeister, Christoph Wierling, Axel Kowald, and Ralf Herwig. *Systems biology: a textbook*. John Wiley & Sons, 2016.

[39] Libor Krásnỳ and Richard L Gourse. An alternative strategy for bacterial ribosome synthesis: Bacillus subtilis rrna transcription regulation. *The EMBO journal*, 23(22):4473–4483, 2004.

[40] Allison Kriel, Alycia N Bittner, Sok Ho Kim, Kuanqing Liu, Ashley K Tehranchi, Winnie Y Zou, Samantha Rendon, Rui Chen, Benjamin P Tu, and Jue D Wang. Direct regulation of gtp homeostasis by (p) ppgpp: a critical component of viability and stress resistance. *Molecular cell*, 48(2):231–241, 2012.

[41] Jennifer F Kugel and James A Goodrich. An rna transcriptional regulator templates its own regulatory rna. *Nature chemical biology*, 3(2):89–90, 2007.

[42] Denis LJ Lafontaine and David Tollervey. The function and synthesis of ribosomes. *Nature reviews. Molecular cell biology*, 2(7):514, 2001.

[43] Paul S Lovett and Elizabeth J Rogers. Ribosome regulation by the nascent peptide. *Microbiological reviews*, 60(2):366–385, 1996.

[44] Sergey Melnikov, Adam Ben-Shem, Nicolas Garreau De Loubresse, Lasse Jenner, Gulnara Yusupova, and Marat Yusupov. One core, two shells: bacterial and eukaryotic ribosomes. *Nature structural & molecular biology*, 19(6):560–567, 2012.

[45] Enrique Merino and Charles Yanofsky. Transcription attenuation: a highly conserved regulatory strategy used by bacteria. *Trends in genetics*, 21(5):260–264, 2005.

[46] Houra Merrikh, Yan Zhang, Alan D Grossman, and Jue D Wang. Replication-transcription conflicts in bacteria. *Nature reviews. Microbiology*, 10(7):449, 2012.

[47] Pohl Milón, Cristina Maracci, Liudmila Filonava, Claudio O Gualerzi, and Marina V Rodnina. Real-time assembly landscape of bacterial 30s translation initiation complex. *Nature structural & molecular biology*, 19(6):609–615, 2012.

[48] Magali Naville and Daniel Gautheret. Transcription attenuation in bacteria: theme and variations. *Briefings in Functional Genomics and Proteomics*, 8(6):482–492, 2009.

[49] Knud H Nierhaus. The assembly of prokaryotic ribosomes. *Biochimie*, 73(6):739–755, 1991.

[50] Evgeny Nudler. Rna polymerase backtracking in gene regulation and genome instability. *Cell*, 149(7):1438–1445, 2012.

[51] Sabine Petry, Albert Weixlbaumer, and V Ramakrishnan. The termination of translation. *Current opinion in structural biology*, 18(1):70–77, 2008.

[52] Benjamin A Pierce. *Genetics: A conceptual approach.* Macmillan, 2012.

[53] V Ramakrishnan. Ribosome structure and the mechanism of translation. *Cell*, 108(4):557–572, 2002.

[54] Reinhard Rauhut and Gabriele Klug. mrna degradation in bacteria. *FEMS microbiology reviews*, 23(3):353–370, 1999.

[55] Andrey Revyakin, Chenyu Liu, Richard H Ebright, and Terence R Strick. Abortive initiation and productive initiation by rna polymerase involve dna scrunching. *Science*, 314(5802):1139–1143, 2006.

[56] Andrew Robinson and Antoine M Van Oijen. Bacterial replication, transcription and translation: mechanistic insights from single-molecule biochemical studies. *Nature reviews. Microbiology*, 11(5):303, 2013.

[57] Marina V Rodnina and Wolfgang Wintermeyer. Ribosome fidelity: trna discrimination, proofreading and induced fit. *Trends in biochemical sciences*, 26(2):124–130, 2001.

[58] Peter Russel. *iGenetics: A Molecular Approach.* Benjamin Cummings, 2009.

[59] Ruth M Saecker, M Thomas Record, et al. Mechanism of bacterial transcription initiation: Rna polymerase-promoter binding, isomerization to initiation-competent open complexes, and initiation of rna synthesis. *Journal of molecular biology*, 412(5):754–771, 2011.

[60] Zahra Shajani, Michael T Sykes, and James R Williamson. Assembly of bacterial ribosomes. *Annual review of biochemistry*, 80:501–526, 2011.

[61] Anna V Sherwood and Tina M Henkin. Riboswitch-mediated gene regulation: novel rna architectures dictate gene expression responses. *Annual review of microbiology*, 70:361–374, 2016.

[62] Larry Snyder, Wendy Champness, and Wendy Champness. *Molecular genetics of bacteria.* Number 572.8 S6M6 2007. ASM Press Washington, DC, 2007.

[63] Anjana Srivatsan and Jue D Wang. Control of bacterial transcription, translation and replication by (p) ppgpp. *Current opinion in microbiology*, 11(2):100–105, 2008.

[64] Thomas A Steitz. A structural understanding of the dynamic ribosome machine. *Nature reviews. Molecular cell biology*, 9(3):242, 2008.

[65] Benedikt Steuten, Philipp G Hoch, Katrin Damm, Sabine Schneider, Karen Köhler, Rolf Wagner, and Roland K Hartmann. Regulation of transcription by 6s rnas: Insights from the escherichia coli and bacillus subtilis model systems. *RNA biology*, 11(5):508–521, 2014.

[66] Jonathan M Stokes and Eric D Brown. Chemical modulators of ribosome biogenesis as biological probes. *Nature chemical biology*, 11(12):924, 2015.

[67] Kozo Tomita and Seisuke Yamashita. Molecular mechanisms of template-independent rna polymerization by trna nucleotidyltransferases. *Frontiers in genetics*, 5, 2014.

[68] Charles L Turnbough and Robert L Switzer. Regulation of pyrimidine biosynthetic gene expression in bacteria: repression without repressors. *Microbiology and Molecular Biology Reviews*, 72(2):266–300, 2008.

[69] Serafín Vivanco-Domínguez, José Bueno-Martínez, Gloria León-Avila, Nobuhiro Iwakura, Akira Kaji, Hideko Kaji, and Gabriel Guarneros. Protein synthesis factors (rf1, rf2, rf3, rrf, and tmrna) and peptidyl-trna hydrolase rescue stalled ribosomes at sense codons. *Journal of molecular biology*, 417(5):425–439, 2012.

[70] Margaritis Voliotis, Netta Cohen, Carmen Molina-París, and Tanniemola B Liverpool. Backtracking and proofreading in dna transcription. *Physical review letters*, 102(25):258101, 2009.

[71] Peter H von Hippel. An integrated model of the transcription complex in elongation, termination, and editing. *Science*, 281(5377):660–665, 1998.

[72] Karen M Wassarman. Small rnas in bacteria: diverse regulators of gene expression in response to environmental changes. *Cell*, 109(2):141–144, 2002.

[73] Lauren S Waters and Gisela Storz. Regulatory rnas in bacteria. *Cell*, 136(4):615–628, 2009.

[74] Wade C Winkler and Ronald R Breaker. Regulation of bacterial gene expression by riboswitches. *Annu. Rev. Microbiol.*, 59:487–517, 2005.

[75] Helen Yakhnin, Alexander V Yakhnin, and Paul Babitzke. Translation control of trpg from transcripts originating from the folate operon promoter of bacillus subtilis is influenced by translation-mediated displacement of bound trap, while translation control of transcripts originating from a newly identified trpg promoter is not. *Journal of bacteriology*, 189(3):872–879, 2007.

[76] Hiroshi Yamamoto, Yan Qin, John Achenbach, Chengmin Li, Jaroslaw Kijek, Christian MT Spahn, and Knud H Nierhaus. Ef-g and ef4: translocation and back-translocation on the bacterial ribosome. *Nature reviews. Microbiology*, 12(2):89, 2014.

[77] Charles Yanofsky. Transcription attenuation: once viewed as a novel regulatory strategy. *Journal of Bacteriology*, 182(1):1–8, 2000.

[78] Charles Yanofsky. Rna-based regulation of genes of tryptophan synthesis and degradation, in bacteria. *Rna*, 13(8):1141–1154, 2007.

[79] Nikolay Zenkin and Yulia Yuzenkova. New insights into the functions of transcription factors that bind the rna polymerase secondary channel. *Biomolecules*, 5(3):1195–1209, 2015.

[80] Ekaterina Zhilina, Daria Esyunina, Konstantin Brodolin, and Andrey Kulbachinskiy. Structural transitions in the transcription elongation complexes of bacterial rna polymerase during σ-dependent pausing. *Nucleic acids research*, 40(7):3078–3091, 2011.
