# Supplementary figures and images for: The bacterial interlocked process ONtology (BiPON): a systemic multi-scale unified representation of biological processes in prokaryotes
Source: J Biomed Semantics. 2017 Nov 23;8:53. doi: 10.1186/s13326-017-0165-6 (PMC5701433; doi:10.1186/s13326-017-0165-6)

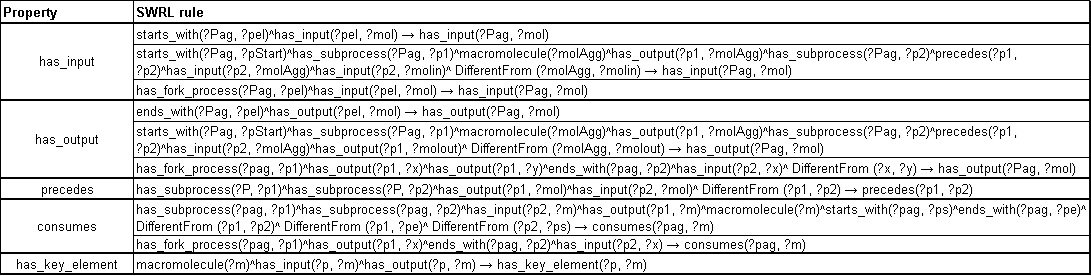

Supplement: Supplementary file 2 — Table of SWRL rules for the properties consumes, has_input, has_output, has_key_element, affects, precedes, before. (TIFF 90 kb) [file 13326_2017_165_MOESM2_ESM.tif]

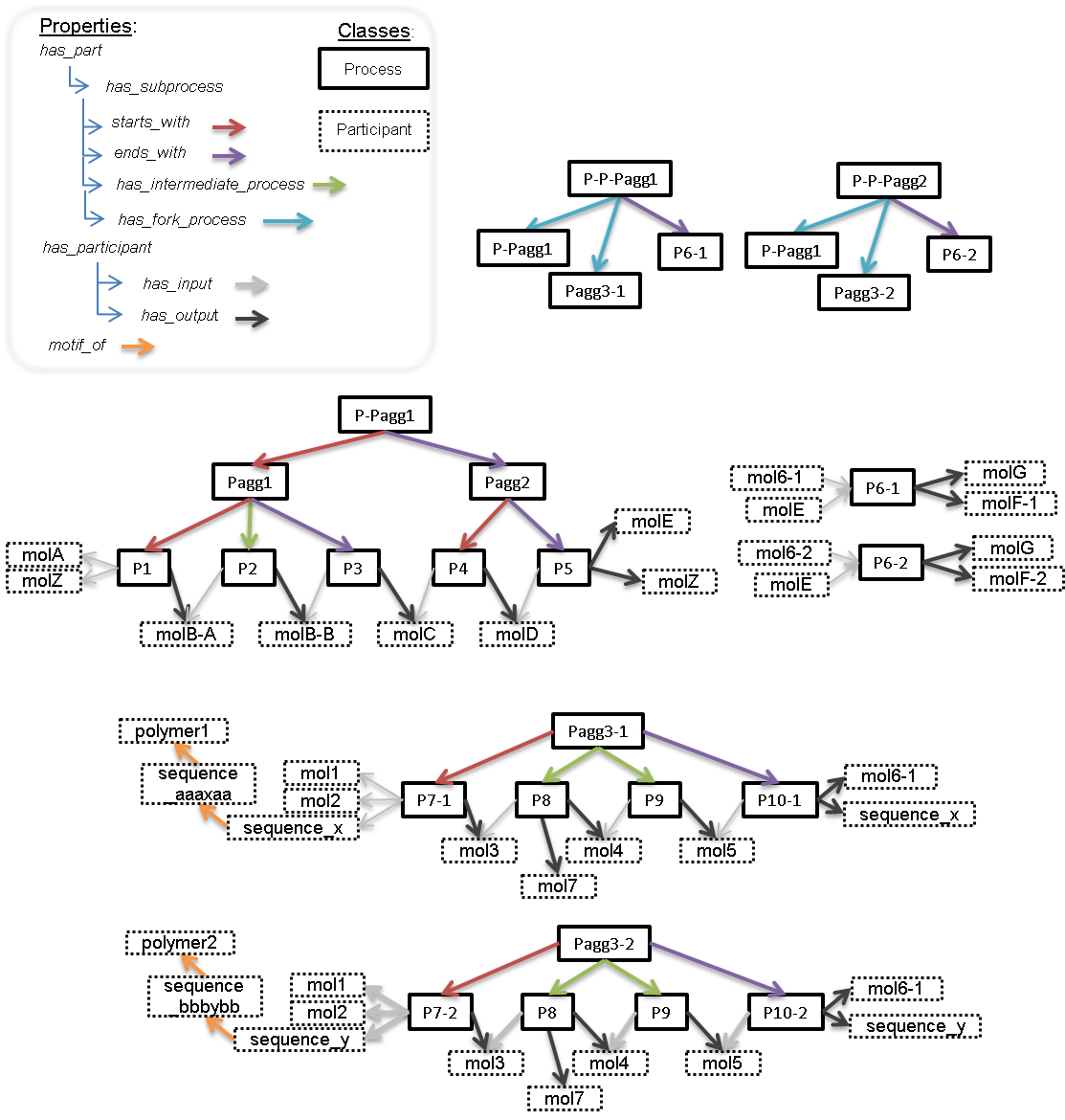

Supplement: Supplementary file 4 — Graphical representation of the biological processes, chemical entities, and properties included in toyBiPON. (PNG 205 kb) [file 13326_2017_165_MOESM4_ESM.png]
